# Supplementary material for: Comparing three approaches to modelling the effects of temperature and rainfall on malaria incidence in different climatic regions
Source: BMC Public Health. 2026 Jan 27;26:674. doi: 10.1186/s12889-026-26280-0 (PMC12918482; doi:10.1186/s12889-026-26280-0)
Supplement: Supplementary file 2 — Supplementary Material 2. [file 12889_2026_26280_MOESM2_ESM.pdf]

Appendix II: Supplemental Plots

Transmission Parameter Curves by Approach

The following functions represent how temperature affects key transmission parameters. For Approach A, the mosquito biting rate (a) is derived from studies on *Anopheles pseudopunctipennis* vectors [1, 26, 44, 45], and the mortality rates (μ_l and μ_m) were defined using data from *Culex quinquefasciatus and Aedes aegypti* mosquitoes [43]. Figure S1 provides plots showing how these parameters change with temperature.

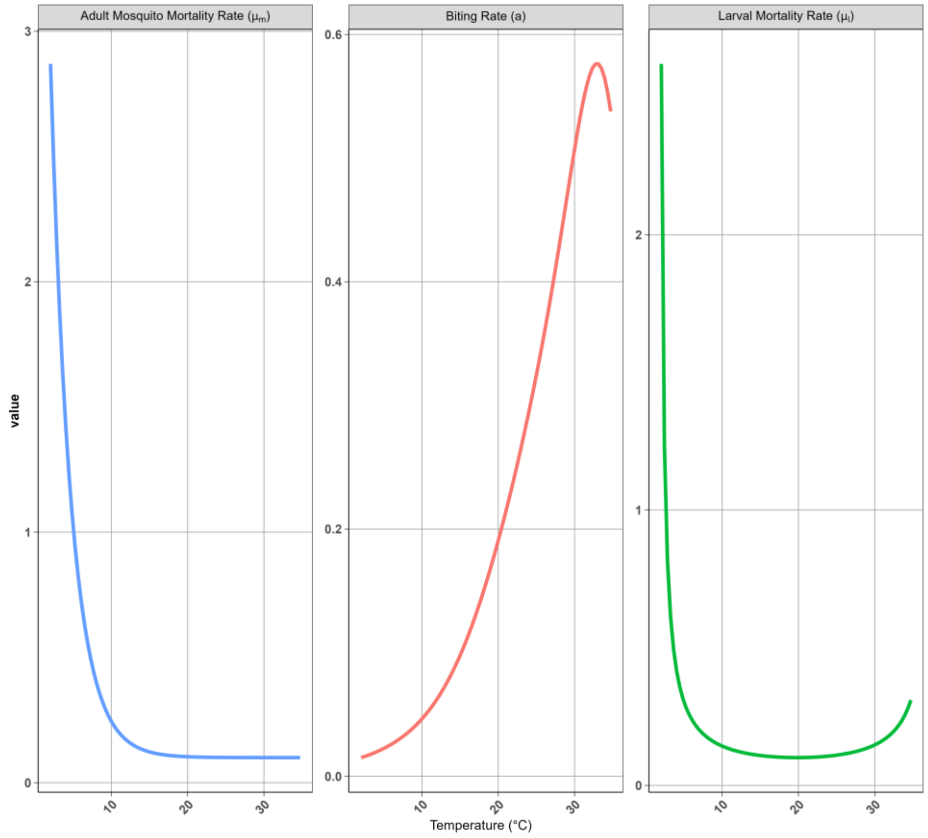

Fig. S1: Model parameters used for Approach A.

Figure S2 shows thermally driven changes in egg production (n_e), oviposition (ϑ), development (κ_e, κ_l, and κ_p), and mortality (μ_e and μ_l), were parameterized for *A. arabiensis* [22, 39] in Approach B. These processes were captured through cubic polynomial functions constrained to biologically plausible values. The mosquito biting

rate (a) follows a non-linear thermal response drawing on empirical relationships for *Anopheles spp.* and *A. pseudopunctipennis* [22, 41].

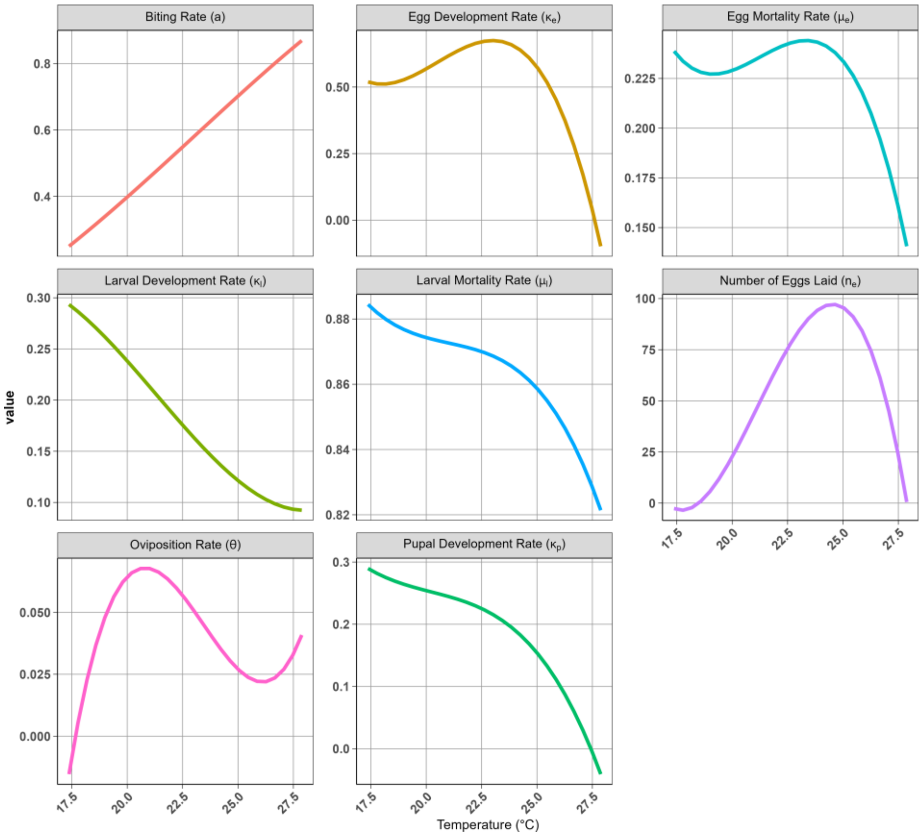

Fig. S2: Model parameters used for Approach B.

Figure S3 shows how in Approach C, the larval mortality rate (μ_l), adult mortality rate (μ_m), parasite development rate (γ_m), and biting rate (a) are defined using the following non-linear thermal functions. These functions were parameterized from studies of *Anopheles spp.* and the *A. gambiae* complex [22, 25, 40, 42], alongside biting rate relationships informed by *A. pseudopunctipennis* [22, 41].

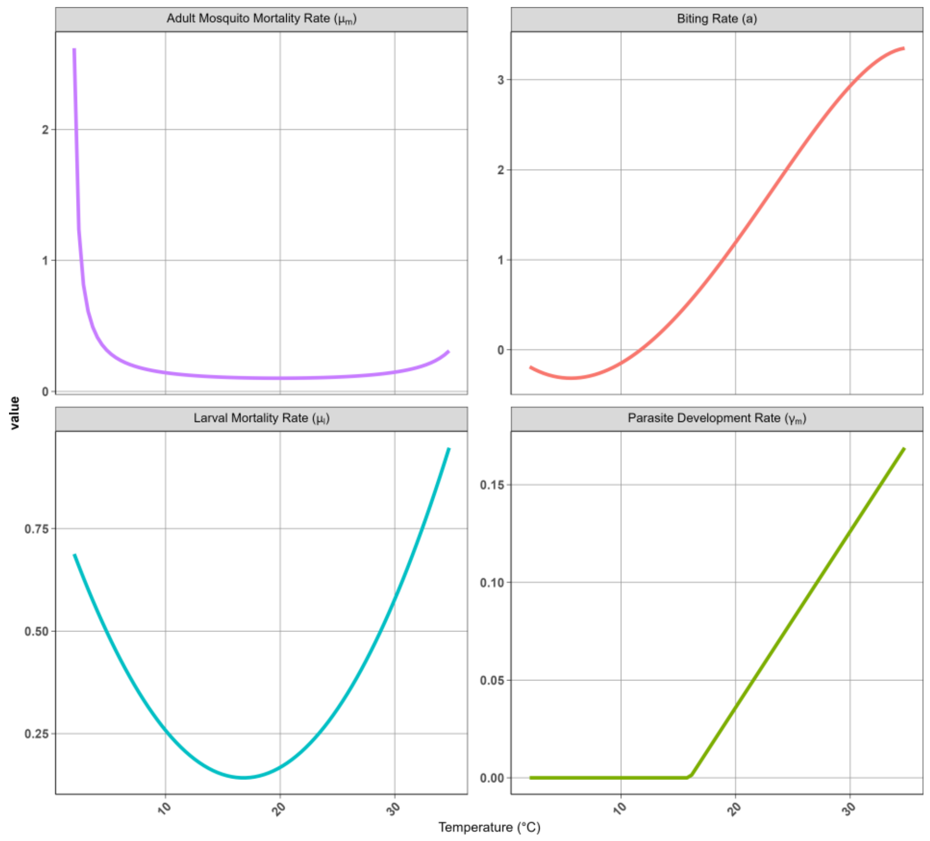

Fig. S3: Model parameters used for Approach C.

Surface Responses to Temperature and Rainfall (Approach C)

Figure S4 shows that the carrying capacity (K_m) for mosquito breeding increases linearly with rainfall, reflecting resource availability.

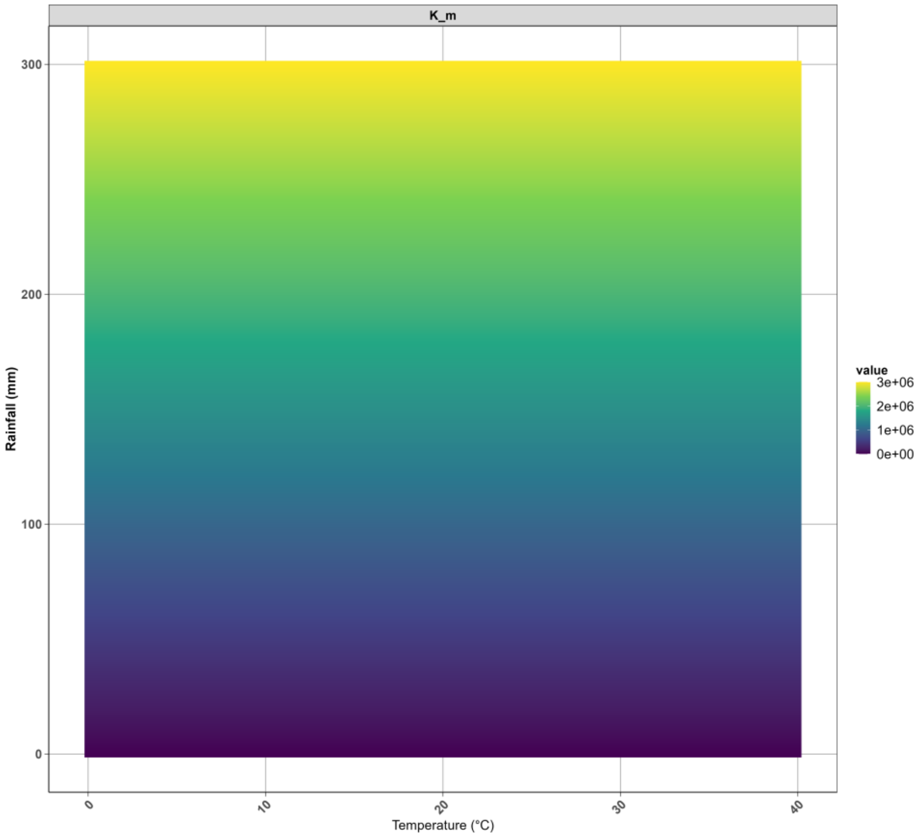

Fig. S4: Adult carrying capacity profile used for Approach C.

Surface plots of egg, larval, and pupal development rates (κ_e, κ_l, and κ_p) derived from studies on *A. gambiae* complex mosquitoes show complex interactions between climatic variables under Approach C [22, 25, 40, 42].

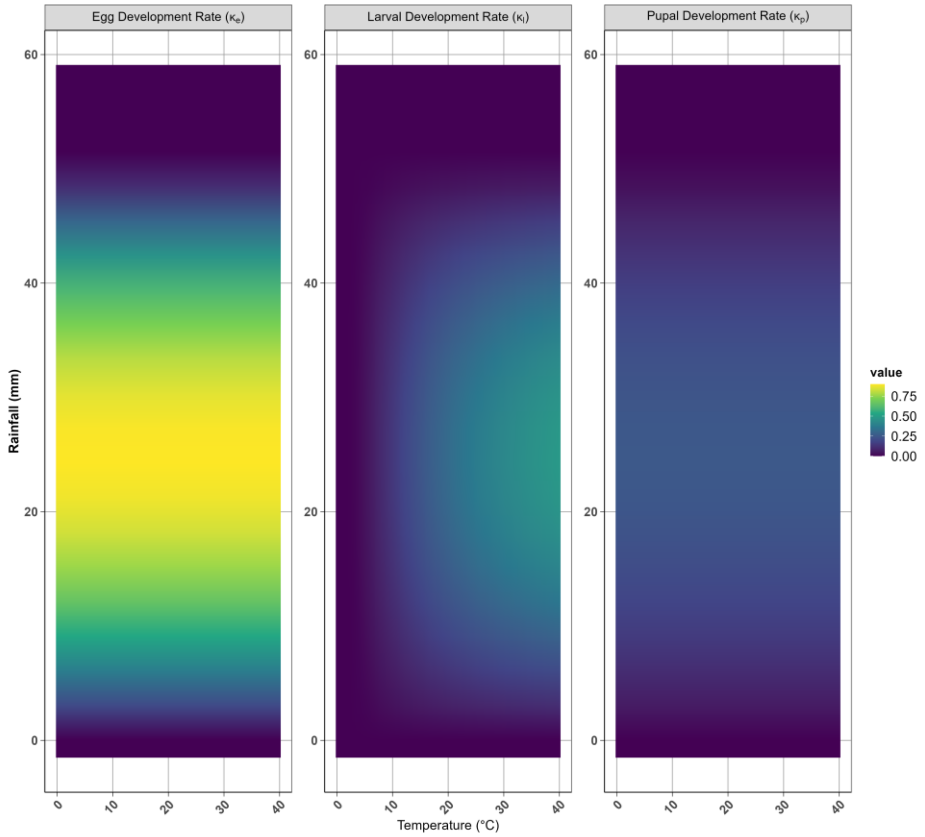

Fig. S5: Survival rate profiles for eggs, larvae and pupae used for Approach C.

Table S2: Summary of functional forms used in each approach.

Symbol	Description	Function	Approach
a	Adult mosquito biting rate	$\frac{1.25}{107.204-13.3523T+0.677509T^2-0.0159732T^3+0.000144876T^4}$	A
μ_l	Larval mortality rate	$\max\left(0, \frac{1}{-4.4+1.31(T+2)-0.03(T+2)^2}\right)$	A
μ_m	Mortality rate in adult mosquitoes	$\frac{3.04}{30.4}+\frac{29.564}{30.4}\times\exp\left(-\frac{T+273.15-278}{2.7035}\right)$	A
a	Adult mosquito biting rate	$2.5\times 0.000203\times(T^2-11.7T)\times\sqrt{42.3-T}$	B
n_e	Total number of eggs laid	$-3.387987+1.038248\times(-0.61411T^3+38.93T^2-801.27T+5391.4)$	B
θ	Egg laying rate	$-7.648455+1.147739\times(0.00054T^3-0.038T^2+0.88T)$	B
κ_e	Egg hatching rate	$-0.3485345-0.2186501\times(0.012(T+2)^3-0.81(T+2)^2+18(T+2)-135.93)$	B
μ_e	Egg mortality rate	$0.2874394-0.1396794\times(0.0033(T+2)^3-0.23(T+2)^2+5.3(T+2)-40)$	B
κ_l	Larval develop-ment rate	$0.4722003-0.09495794\times(-0.002(T+2)^3+0.14(T+2)^2-3(T+2)+22)$	B
μ_l	Larval mortality rate	$1.115093-0.1583374\times(0.00081(T+2)^3-0.056(T+2)^2+1.3(T+2)-8.6)$	B
κ_p	Pupal devel-opment rate	$0.3881068+0.2755463\times(-0.0018(T+2)^3+0.12(T+2)^2-2.7(T+2)+20)$	B

(Continues on the next page)

(Continued from the previous page)

a	Adult mosquito biting rate	$7.5 \times 0.000203 \times (T^2 - 11.7T) \times \sqrt{42.3 - T}$	C
K_m	Adult mosquito carrying capacity	$10^4 \times R$	C
κ_e	Egg hatching rate	$\min\left(1, \frac{4 \times \max p_e}{R_l^2} \times R \times \max(0, R_l - R)\right)$	C
t_l	Larval development period	$0.0557(T+2) - 0.06737$	C
κ_l	Larval development rate	$\exp\left(-\frac{1}{t_l}\right) \times \min\left(1, \frac{4 \times \max p_l}{R_l^2} \times R \times \max(0, R_l - R)\right)$	C
κ_p	Pupal development rate	$\min\left(1, \frac{4 \times \max p_p}{R_l^2} \times R \times \max(0, R_l - R)\right)$	C
μ_l	Larval mortality rate	$0.0025(T+2)^2 - 0.094(T+2) + 1.0257$	C
μ_m	Mortality rate in adult mosquitoes	$\max\left(0, \frac{1}{-4.4 + 1.31T - 0.03T^2}\right)$	C
γ_m	Parasite development rate in infected mosquitoes	$\max\left(0, \frac{T - 16}{111}\right)$	C

Climate Data

Figure S7 provides a timeseries of mean monthly temperature trends from the Climate Change Knowledge Portal (CCKP) are shown across the semi-arid, tropical, and sub-tropical regions.

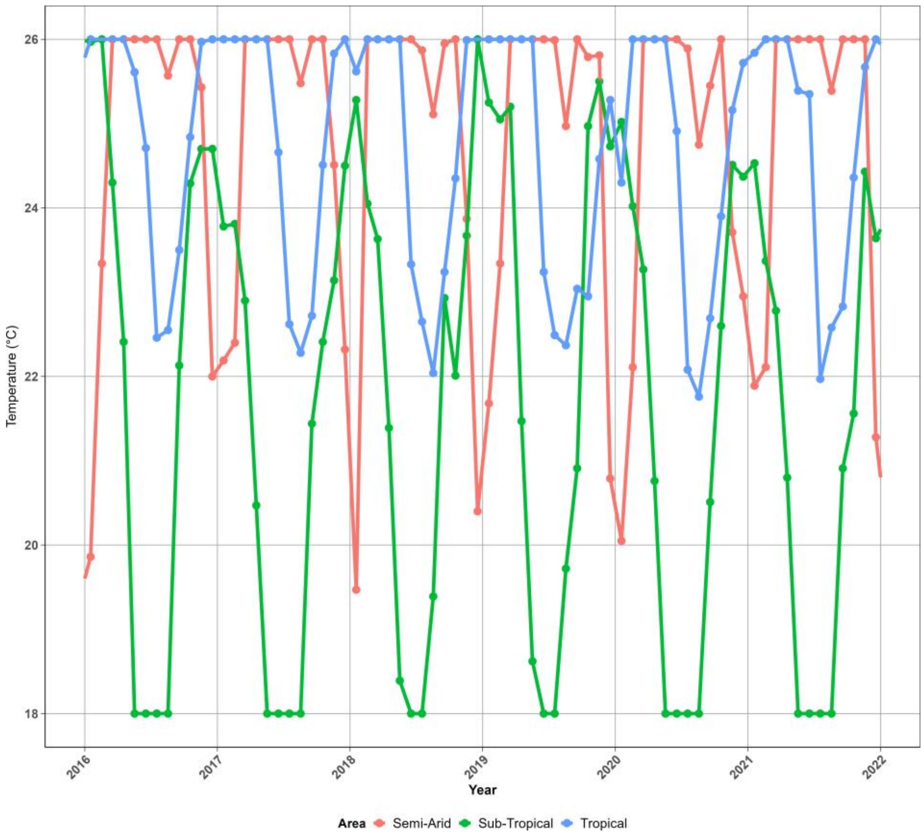

Fig. S7: Temperature trends for the 2016 to 2022 period

Figure S8 provides a timeseries of mean monthly rainfall trends from the Climate Change Knowledge Portal (CCKP) are shown across the semi-arid, tropical, and sub-tropical regions.

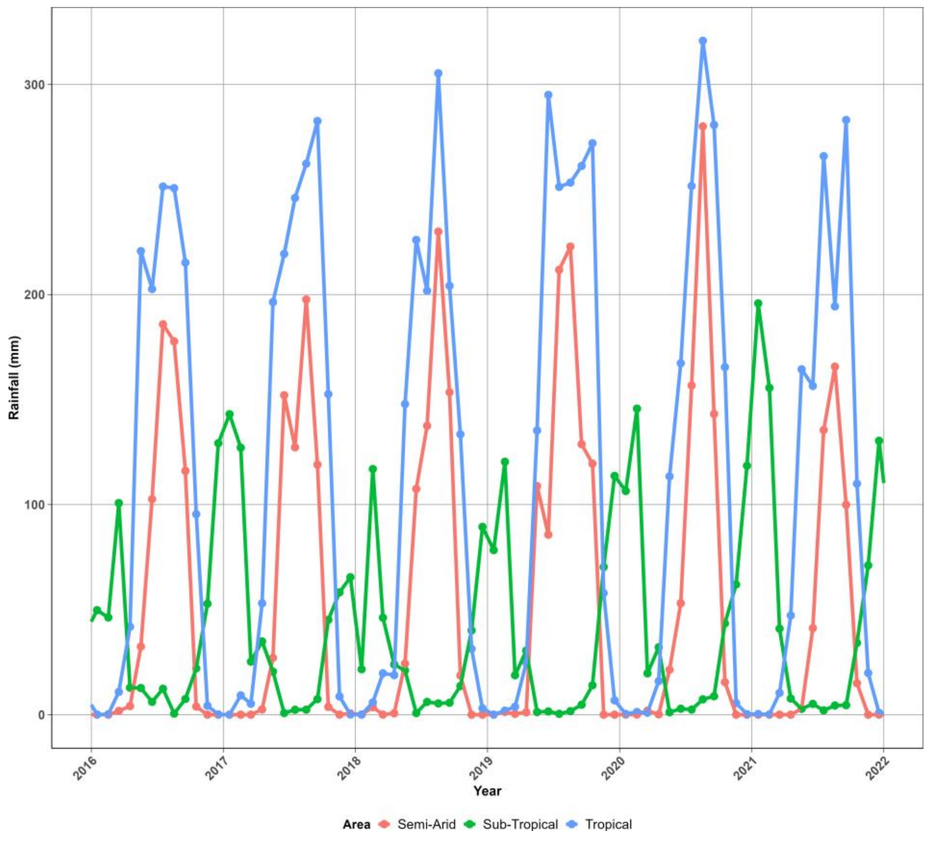

Fig. S8: Rainfall trends for the 2016 to 2022 period

Time-Series of Derived Parameters by Approach

Temperature-derived values of biting rate and mortality over time across semi-arid, tropical, and sub-tropical regions.

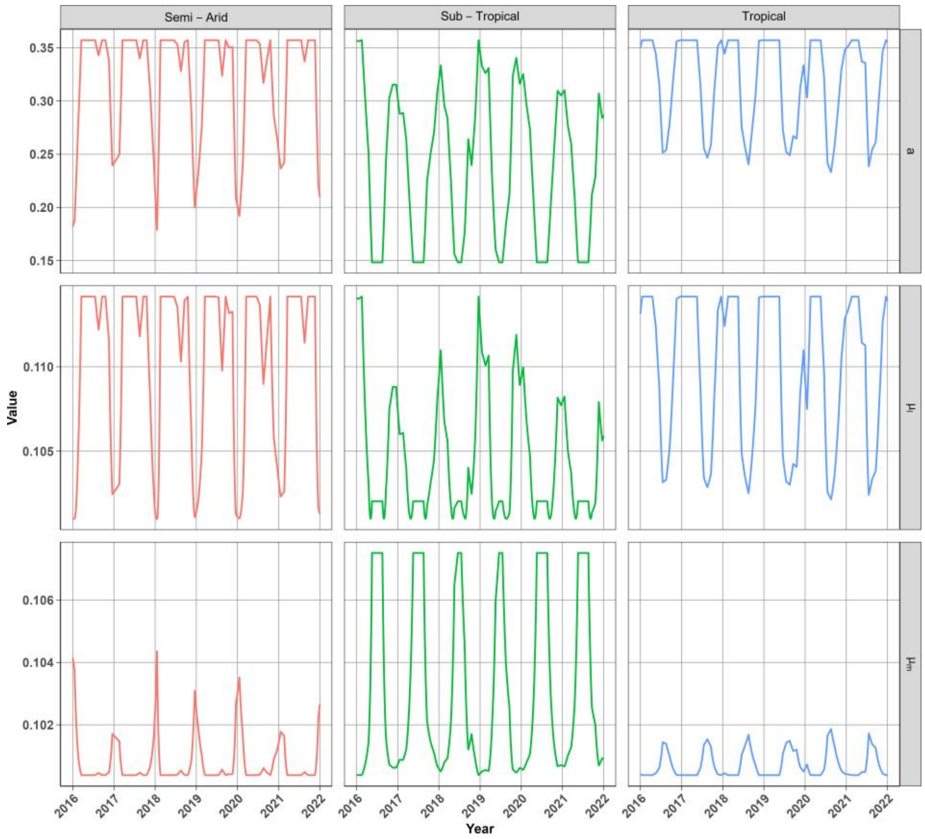

Fig. S9: Model parameters used for Approach A.

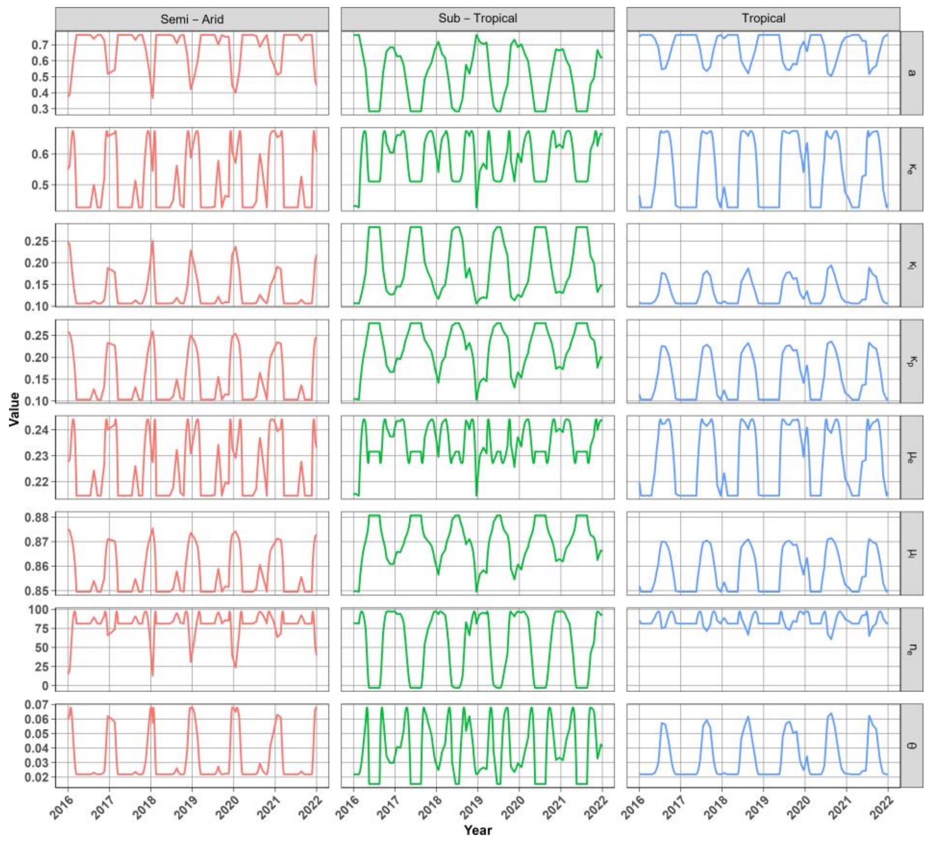

Fig. S10: Model parameters used for Approach B.

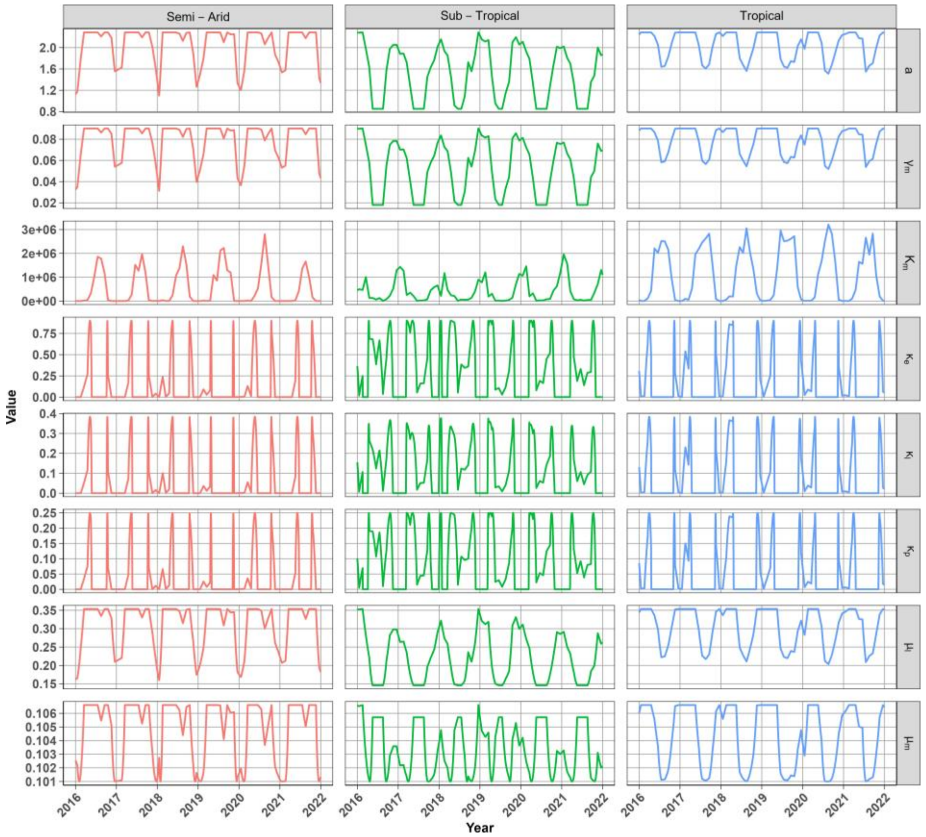

Fig. S11: Model parameters used for Approach C.
